# Troponin T/I discordance in immune checkpoint inhibitor myositis in a patient with ischaemic cardiac disease: a case report

**DOI:** 10.1093/ehjcr/ytag216

**Published:** 2026-03-18

**Authors:** Ali El-Hisnawi, Giuseppe D'Ancona, Helmut Heinze, Robert Steinicke, Hüseyin Ince

**Affiliations:** Department of Cardiology, Vivantes Klinikum Neukölln, Rudower Straße 48, Berlin 12351, Germany; Department of Cardiology, Vivantes Klinikum Am Urban, Dieffenbachstraße 1, Berlin 10967, Germany; Department of Cardiology, General Internal, Medicine and Conservative Intensive Care Medicine, Dieffenbachstraße 1, 10967 Berlin-Kreuzberg, Germany; Department and Outpatient Clinic of Cardiology, Ernst-Heydemann-Straße 6, 18057 Rostock, Germany; Department of Cardiology, Vivantes Klinikum Neukölln, Rudower Straße 48, Berlin 12351, Germany; Department of Cardiology, Vivantes Klinikum Neukölln, Rudower Straße 48, Berlin 12351, Germany; Department of Cardiology, Vivantes Klinikum Neukölln, Rudower Straße 48, Berlin 12351, Germany; Department of Cardiology, General Internal, Medicine and Conservative Intensive Care Medicine, Dieffenbachstraße 1, 10967 Berlin-Kreuzberg, Germany; Department and Outpatient Clinic of Cardiology, Ernst-Heydemann-Straße 6, 18057 Rostock, Germany

**Keywords:** Troponin I, Troponin T, Immune checkpoint inhibitors (ICIs), Myocarditis, Myositis, Acute coronary syndrome, Case report

## Abstract

**Background:**

Immune checkpoint inhibitors (ICIs) are increasingly used in oncology and can cause immune-related adverse events, including cardiotoxicity. Discrepancies between cardiac troponin T (cTnT) and cardiac troponin I (cTnI) have been described in ICI-associated myositis with otherwise normal cardiac findings and may help differentiate myositis from myocarditis. In this case, cTnT elevation was first detected on routine troponin testing performed because myocarditis is a recognized immune-related adverse event of nivolumab, despite the absence of typical cardiac symptoms.

**Case summary:**

A 72-year-old man receiving nivolumab developed marked cTnT elevation. He was referred to cardiology for suspected ICI-related myocarditis. Cardiac MRI was unremarkable. Because of cardiovascular risk factors and unexplained troponinaemia, coronary angiography was performed and revealed an 80% stenosis of the LAD, which was treated with a drug-eluting stent. Post-intervention, cTnT continued to rise while cTnI remained normal on repeated measurements. Differential diagnoses included myositis, myocarditis, and in-stent thrombosis. Taken together, the clinical, imaging, and biomarker findings, in particular the troponin T/I discrepancy, made a cardiac cause of cTnT elevation unlikely. Immune-mediated myositis was confirmed, and mycophenolate mofetil therapy led to clinical and biochemical improvement.

**Conclusion:**

This case may expand current knowledge by showing that, in an ICI-treated patient, troponin T/I discrepancy may not only help differentiate myositis from myocarditis but also assist in distinguishing skeletal muscle involvement from other cardiac causes, including ACS and in-stent thrombosis. Recognition of this pattern may improve diagnostic accuracy and guide appropriate therapeutic strategies in oncological patients presenting with troponinaemia.

Learning points

- Markedly elevated cTnT with normal cTnI in ICI-treated patients may indicate skeletal muscle involvement (myositis) rather than isolated myocardial injury.
- cTnT/cTnI discordance is a useful diagnostic clue but does not exclude myocarditis, ACS, or in-stent thrombosis; interpretation requires careful clinical evaluation, ECG assessment, imaging, and additional biomarkers.

## Introduction

Cardiac troponin T (cTnT) and troponin I (cTnI) are well-established biomarkers routinely used for the diagnosis of myocardial injury of any origin. In clinical practice, both markers are generally considered equivalent due to their high myocardial specificity. However, marked discordance between the plasma levels of these two biomarkers may occur in certain clinical settings, particularly in the presence of skeletal muscle disease. For example, troponin T may be substantially elevated, while troponin I remains within the normal range in inflammatory myopathies.^1^ To date, such findings have only been described in isolated case reports, making evidence-based interpretation challenging. The pathophysiological basis for this discrepancy remains unclear and poses a diagnostic dilemma. Such discordance may lead to uncertainty in clinical decision-making and necessitate careful interpretation of all findings in the specific clinical context. Previous case reports have described instances in which an elevated troponin T level led to suspicion of myocarditis, which was subsequently ruled out based on a normal troponin I result.^2^ In the present report, we describe a patient receiving immune checkpoint inhibitor (ICI) therapy who had three-vessel coronary artery disease and marked troponin T/I discordance, in whom myocarditis, acute coronary syndrome (ACS), and in-stent thrombosis were ultimately ruled out. This case illustrates the potential value of troponin T/I discrepancy as a tool to differentiate skeletal muscle from myocardial injury in a complex inflammatory and ischaemic setting.

## Summary figure

**Figure:**
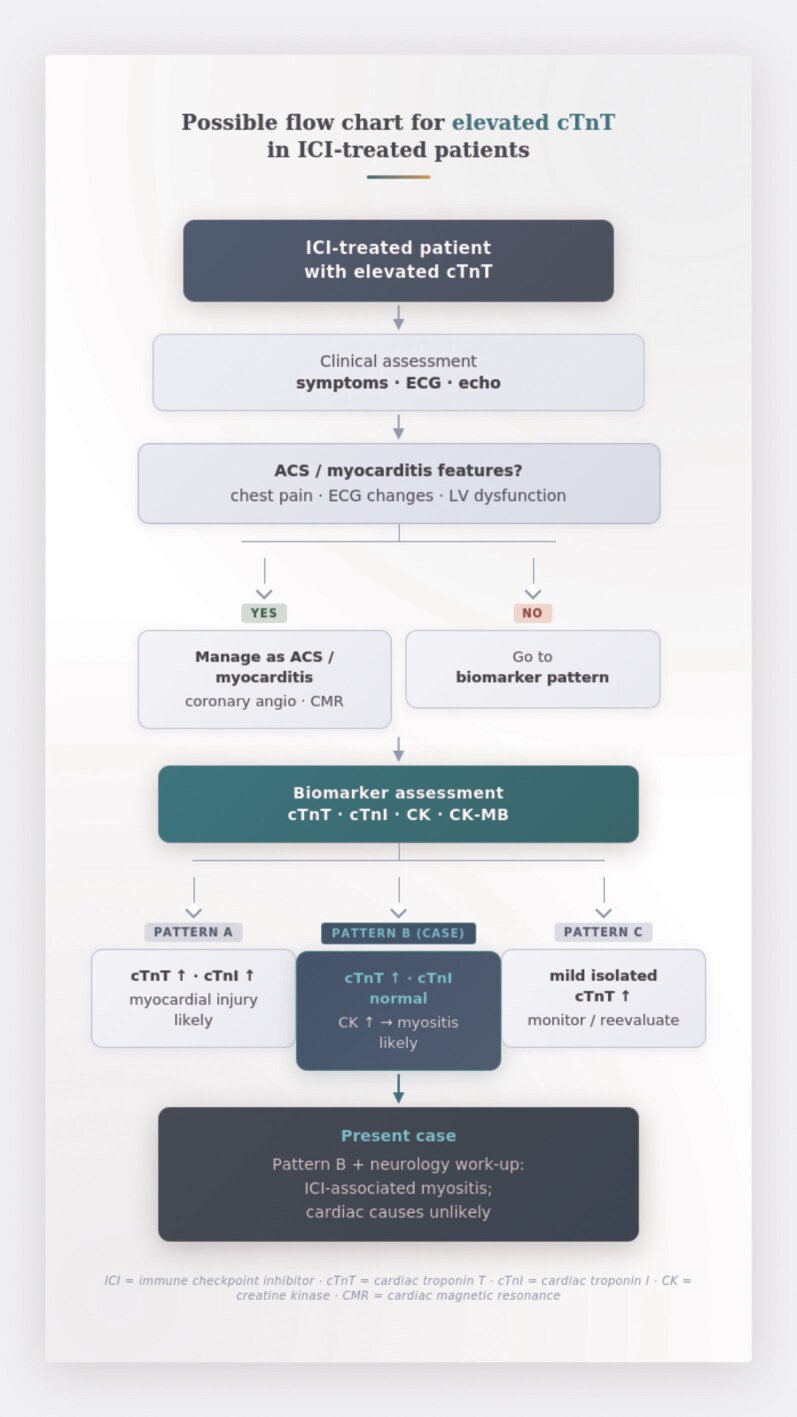


## Case presentation

A 72-year-old man presenting with fatigue was admitted to our cardiology department for evaluation of elevated troponin T levels in the context of suspected autoimmune myocarditis. Troponin T had initially been measured on the dermatology ward as part of routine monitoring, given the adverse effect profile of nivolumab, which includes immune-mediated myocarditis. Laboratory findings had already raised suspicion of autoimmune thyroiditis and autoimmune hepatitis, both recognized immune-related adverse events of nivolumab, raising concern for the development of multiple autoimmune complications in this patient. His medical history included malignant melanoma in the right thigh, obstructive sleep apnoea syndrome (OSAS), type 2 diabetes mellitus, and arterial hypertension. There was no evidence of renal insufficiency.

**Figure 1:**
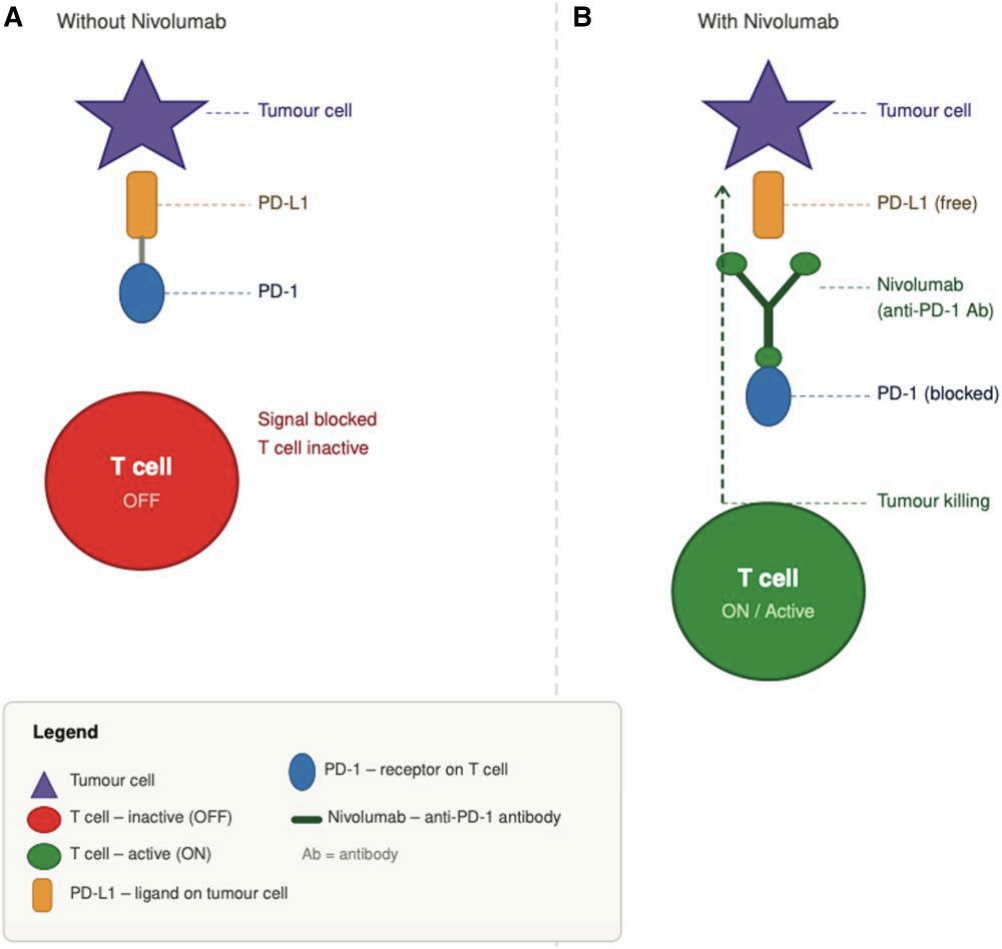
Mechanism of action of nivolumab.

One month earlier, the patient had undergone surgical resection of the melanoma on the dermatology ward and had received his first dose of nivolumab, a PD-1 receptor inhibitor used as immunotherapy in various malignancies (see *Figure 1* for mechanism of action of nivolumab). On admission, the patient reported marked fatigue; myalgias predominantly affecting the neck, shoulders, and thighs; and a sensation of heaviness and tremor in the legs after minimal exertion. He denied chest pain, anginal symptoms, dyspnoea, pleuritic chest pain, syncope, and haemoptysis. Initial laboratory testing showed a markedly elevated creatine kinase (CK) level of 8341 U/L (reference <190 U/L), with a CK-MB fraction of 27% (reference <6%), and a substantially elevated troponin T level of 754 ng/L (reference <14 ng/L). NT-proBNP was within the normal range.

**Figure 2:**
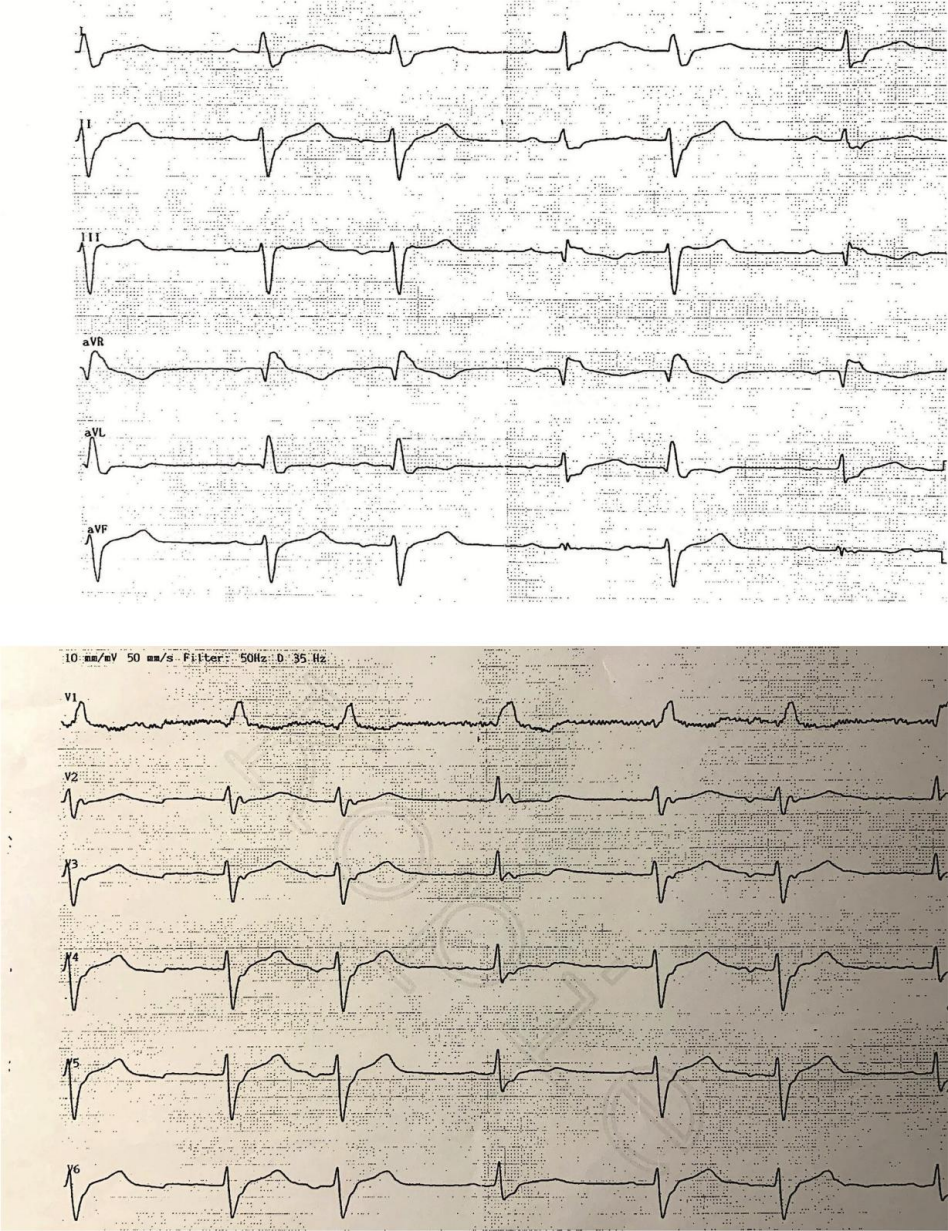
Admission 12-lead electrocardiogram. 12-lead ECG showing first-degree atrioventricular block, right bundle branch block, and left anterior hemiblock, without signs of acute myocardial ischaemia.

Electrocardiography showed a known right bundle branch block (RBBB) and left anterior hemiblock (LAHB), with secondary repolarization abnormalities but no ischaemic changes. QT and PR intervals were within normal limits, while QRS duration was prolonged at 162 ms (see *Figure 2*). These ECG abnormalities had been documented before dermatological treatment and prior to the initiation of nivolumab. Echocardiography demonstrated normal cardiac chamber sizes, preserved left ventricular ejection fraction (>51%), and normal biventricular wall motion without significant valvular pathology. The interventricular septum was mildly thickened (11 mm). As the RBBB was pre-existing, the patient had no specific symptoms suggestive of pulmonary embolism, and transthoracic echocardiography showed no signs of right ventricular strain—D-dimer testing and CT pulmonary angiography were not pursued.

**Figure 3:**
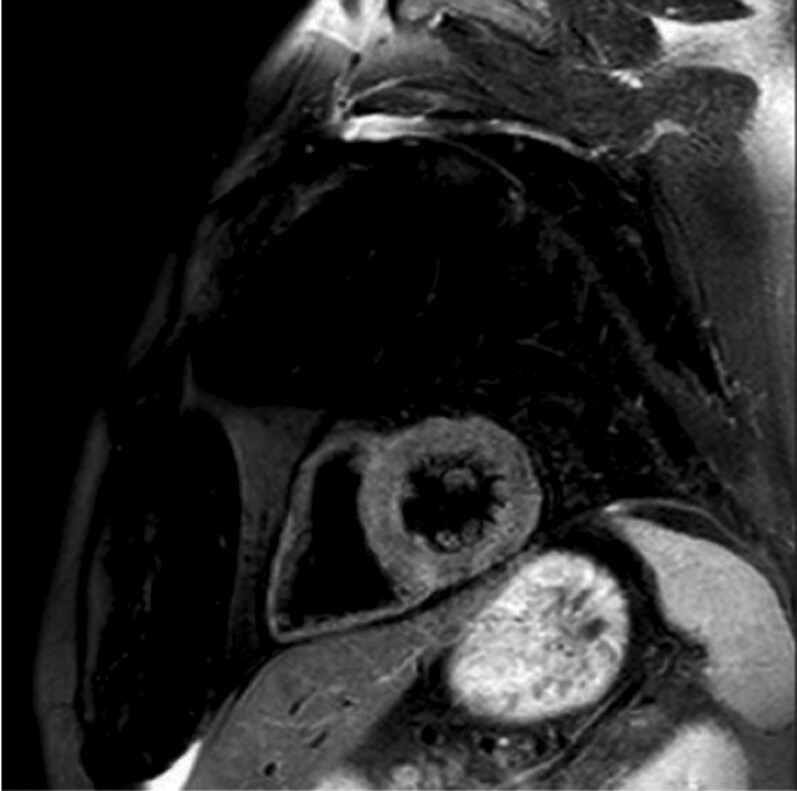
Cardiac magnetic resonance imaging. Cardiac MRI with T1 and T2 mapping demonstrating preserved biventricular function and no evidence of myocardial oedema, fibrosis, necrosis, or late gadolinium enhancement according to the updated Lake Louise Criteria, arguing against active myocarditis.

**Figure 4:**
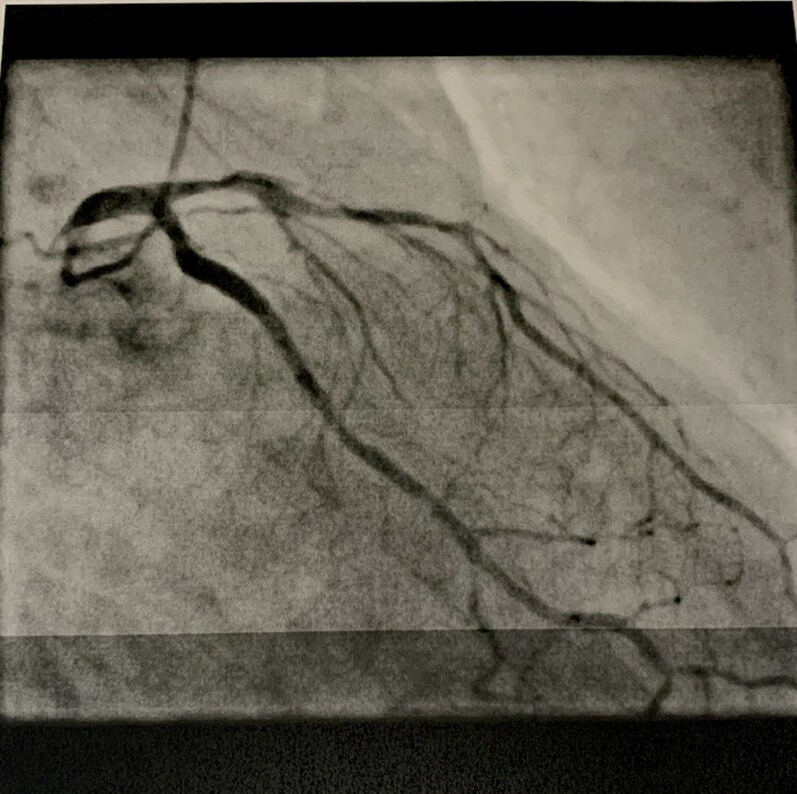
Coronary angiography of the left anterior descending artery. Still frame extracted from the DICOM angiography dataset showing a significant 80% stenosis of the mid left anterior descending artery (Segment 6).

Cardiac MRI was performed because of the high suspicion of checkpoint inhibitor-associated myocarditis. According to the updated Lake Louise Criteria, T1 and T2 mapping were unremarkable, with no evidence of myocardial oedema, fibrosis, necrosis, or scarring (see *Figure 3*). Given the unexplained troponinaemia and the presence of cardiovascular risk factors, coronary angiography was performed. This revealed three-vessel coronary artery disease, including a significant 80% stenosis in the left anterior descending artery (mid-segment or Segment 6), which was treated with implantation of a drug-eluting stent (see *Figure 4*). However, the cause of the troponinaemia remained unclear over the following days. Neither the MRI findings nor the clinical presentation supported myocarditis, and the coronary artery disease did not fulfil criteria for ACS because of the absence of symptoms, ECG changes, and the continued rise in troponin T levels over several days after coronary angiography.

**Figure 5:**
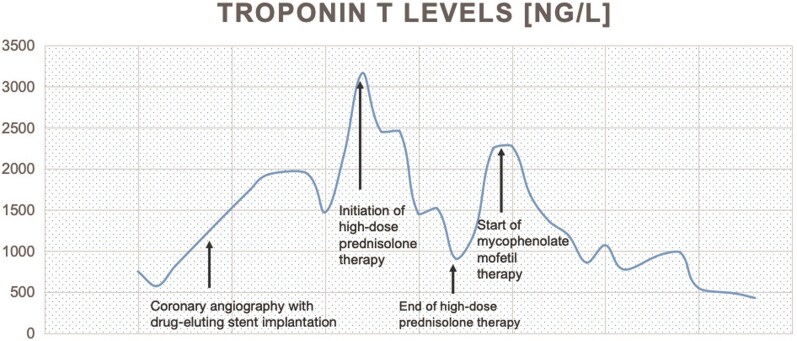
Troponin T time course and its relation to clinical events.

In parallel, neurology was consulted on Day 2 after completion of the initial cardiac work-up, including CMR and PCI, because persistent fatigue and proximal muscle weakness raised suspicion of myositis in the context of the known adverse effect profile of nivolumab. The neurology team performed a targeted clinical examination, assessed CK levels, and carried out electromyography; the findings supported the suspicion of predominantly axial myositis. Because dual antiplatelet therapy had already been established after stent implantation, muscle biopsy was deferred. Prednisolone therapy at a dose of 200 mg/day had already been started on the dermatology ward before transfer to the cardiology ward because of suspected nivolumab-associated autoimmune diathesis, including thyroiditis and hepatitis. Following neurological consultation, prednisolone at 200 mg/day was initially continued and then escalated to 1 g/day for 5 days, starting on Day 8 after coronary angiography because of persistent muscle weakness and only partial improvement in CK levels. After completion of high-dose prednisolone therapy, the dose was reduced again to 200 mg/day and then tapered gradually over the following weeks. Although CK levels decreased after prednisolone treatment, they remained elevated and clinical improvement was limited. Because steroid-refractory myositis was suspected, immunosuppressive therapy with mycophenolate mofetil (1 g twice daily) was initiated after tapering corticosteroids back to 200 mg/day. Before escalation to high-dose prednisolone and initiation of mycophenolate mofetil therapy, serial measurements showed progressively increasing troponin T levels, peaking at 3166 ng/L on Day 8 after coronary angiography, representing more than a four-fold increase from baseline (see *Figure 5*).

Given the markedly elevated cTnT level, the absence of imaging or clinical evidence of myocardial injury, and the clinical suspicion of myositis as an immune-related adverse event of nivolumab, we deliberately requested measurement of cTnI using an alternative high-sensitivity assay before escalation of immunosuppressive therapy with mycophenolate mofetil. This was done because several publications in patients with myositis have described elevated cTnT in the presence of normal cTnI, supporting a skeletal muscle rather than a myocardial origin of cTnT elevation.^1^

In contrast to cTnT, cTnI remained within the normal range (0.02 ng/mL) on five separate measurements during the hospital stay. Despite persistently elevated troponin T levels, no new symptoms developed, in particular, no cardiac symptoms, and repeat ECG, echocardiography, and cardiac MRI remained unchanged. Dual antiplatelet therapy (aspirin 100 mg once daily and ticagrelor 90 mg twice daily) was continued without interruption. There was therefore no evidence of in-stent thrombosis or ACS at any time.

Following initiation of mycophenolate mofetil, CK normalized (<190 U/L); troponin T decreased markedly, with values ranging between 162 and 218 ng/L; and the patient’s symptoms improved significantly. Myalgias resolved completely, and he regained full ambulatory capacity. In the absence of cardiac symptoms, with uninterrupted antiplatelet therapy, consistently unremarkable post-interventional imaging including repeated transthoracic echocardiography and ECG, and persistently negative troponin I, a cardiac origin of the elevated troponin T was excluded. The troponinaemia was ultimately attributed to underlying autoimmune myositis.

After discharge from the cardiology ward, the patient returned to our clinic several times for staging of the malignant melanoma. This staging included cardiac MRI; CT of the neck, chest, abdomen, and pelvis; as well as lymph node ultrasound. At no time was there evidence of local recurrence, lymph node involvement, or distant metastases. No alternative oncological therapy was initiated. S100 and LDH levels remained within the normal range throughout. At the most recent CT examination, however, markedly enlarged left supraclavicular lymph nodes were detected, ultimately leading to the diagnosis of chronic lymphocytic leukaemia (CLL).

## Discussion

Previous studies have shown that patients with myopathies of various aetiologies may exhibit elevated troponin T levels in the absence of evidence of cardiomyocyte injury, whereas troponin I typically remains within the normal range.^1^ Although the underlying mechanisms are not yet fully understood, recent publications have proposed re-expression of cTnT in the skeletal muscle as a plausible explanation.^3^ Animal studies have demonstrated that cTnT is physiologically expressed in the skeletal muscle during embryonic and foetal development, whereas troponin I expression is restricted to myocardial tissue.^4^ Postnatal suppression of troponin T expression in the skeletal muscle is a regulated process that may be disrupted in myopathy, resulting in aberrant re-expression of cTnT. In addition, cross-reactivity of certain cTnT assays with skeletal muscle isoforms has been suggested as another mechanism contributing to elevated cTnT levels in patients without overt myocardial damage. Several case reports in patients receiving ICI therapy have also shown that a troponin discrepancy, with elevated troponin T and normal troponin I, may help to rule out myocarditis.^2^

Our case adds to this body of evidence by demonstrating a similar troponin pattern in a patient with ICI-associated myositis and concomitant coronary artery disease, in whom myocarditis, in-stent thrombosis, and other manifestations of ACS were carefully considered and ultimately deemed unlikely. Importantly, we do not propose troponin T/I discordance as a definitive rule-out test. Rather, we regard it as a strong diagnostic clue that should always be interpreted in conjunction with clinical presentation, ECG, imaging findings, and additional biomarkers.

Immune checkpoint inhibitors, including the PD-1 inhibitor nivolumab, are increasingly used across a broad range of malignancies. Although they have significantly improved oncological outcomes, they are associated with immune-related adverse events (irAEs) affecting multiple organ systems, including autoimmune myositis, thyroiditis, hepatitis, myocarditis, and pneumonitis.^5^ ICI-related myocarditis, while rare, is associated with high morbidity and mortality and is frequently accompanied by concurrent myositis and myasthenia-like syndromes. Autoimmune myositis is characterized by proximal muscle weakness, significantly elevated CK levels, and, in some cases, respiratory or bulbar involvement. Diagnostic evaluation typically includes clinical assessment, serum muscle enzymes, myositis autoantibodies, electromyography, muscle MRI, and, in selected cases, muscle biopsy. In our patient, the combination of proximal muscle weakness, markedly elevated CK, and characteristic electromyography findings strongly supported the diagnosis of ICI-associated autoimmune myositis.

At the same time, the case illustrates in detail how myocarditis, ACS, and in-stent thrombosis were excluded. First, repeated cardiac MRI examinations performed according to the updated Lake Louise Criteria showed no evidence of myocardial oedema, hyperaemia, or fibrosis, making clinically relevant myocarditis unlikely. Second, despite a marked and progressive rise in cTnT, cTnI remained within the normal range on repeated measurements, arguing against substantial myocardial injury. Third, the patient never developed typical anginal symptoms, new ischaemic ECG changes, new regional wall motion abnormalities, or any other pathological findings on serial echocardiograms, making ACS improbable. Moreover, dual antiplatelet therapy was maintained without interruption, and there were no clinical events suggestive of stent thrombosis. Finally, only treatment directed at the autoimmune myositis resulted in both clinical improvement and a significant reduction in troponin T levels. Taken together, these findings strongly support a skeletal muscle rather than a myocardial source of cTnT.

At the same time, this case highlights the limitations of current diagnostic tools. Cardiac MRI is highly informative but not infallible; false-negative results may occur in very early or focal myocarditis.^6^ Biomarker kinetics may also be influenced by systemic inflammation, renal function, and assay characteristics. In addition, a normal cTnI level does not absolutely exclude minimal myocardial injury, particularly in the presence of very small or patchy lesions. For these reasons, troponin T/I discordance should not be used in isolation but should be integrated into a comprehensive multidisciplinary assessment.

From a practical perspective, the diagnostic pathway summarized in the central illustration may help clinicians managing ICI-treated patients who present with elevated troponin T levels. In patients with systemic symptoms and markedly elevated CK, the combination of elevated cTnT and normal cTnI should prompt careful consideration of myositis and early neurological involvement while ensuring that life-threatening cardiac conditions such as myocarditis and ACS are appropriately excluded. Prospective studies systematically assessing cTnT and cTnI in patients with ICI-associated myositis and myocarditis, in combination with standardized CMR protocols and, where feasible, histology, may better define the sensitivity, specificity, and limitations of troponin discordance in this population.

## Lead author biography


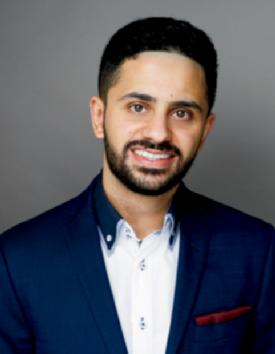


Dr. El-Hisnawi obtained his medical degree at the TU Dresden (Germany). He is a cardiology resident at the Vivantes Neukoelln Hospital in Berlin. Areas of interest include electrophysiology, heart failure, cardiotoxicity, and echocardiography.

## Data Availability

The data underlying this case report are included within the article. Additional de-identified clinical data may be made available from the corresponding author upon reasonable request, in accordance with institutional and data protection regulations; individual patient data are not publicly available in order to protect patient privacy.
